# Obesity Prevention Interventions in US Public Schools: Are Schools Using Programs That Promote Weight Stigma?

**DOI:** 10.5888/pcd14.160605

**Published:** 2017-12-28

**Authors:** Erica L. Kenney, Suzanne Wintner, Rebekka M. Lee, S. Bryn Austin

**Affiliations:** 1 Department of Social and Behavioral Sciences, Harvard T.H. Chan School of Public Health, Boston, Massachusetts; 2 Simmons College School of Social Work, Boston, Massachusetts; 3 Division of Adolescent and Young Adult Medicine, Boston Children’s Hospital, Boston, Massachusetts

## Abstract

**Introduction:**

Despite substantial research on school-based obesity prevention programs, it is unclear how widely they are disseminated. It is also unknown whether schools use obesity programs that inadvertently promote weight stigma or disordered weight-control behaviors.

**Methods:**

In spring 2016, we distributed an online survey about school wellness programming to a simple random sample of US public school administrators (N = 247 respondents; 10.3% response rate). We analyzed survey responses and conducted immersion/crystallization analysis of written open-ended responses.

**Results:**

Slightly less than half (n = 117, 47.4%) of schools offered any obesity prevention program. Only 17 (6.9%) reported using a predeveloped program, and 7 (2.8%) reported using a program with evidence for effectiveness. Thirty-seven schools (15.0%) reported developing intervention programs that focused primarily on individual students’ or staff members’ weight rather than nutrition or physical activity; 28 schools (11.3% of overall) used staff weight-loss competitions. School administrators who reported implementing a program were more likely to describe having a program champion and adequate buy-in from staff, families, and students. Lack of funding, training, and time were widely reported as barriers to implementation. Few administrators used educational (n = 12, 10.3%) or scientific (n = 6, 5.1%) literature for wellness program decision making.

**Conclusion:**

Evidence-based obesity prevention programs appear to be rarely implemented in US schools. Schools may be implementing programs lacking evidence and programs that may unintentionally exacerbate student weight stigma by focusing on student weight rather than healthy habits. Public health practitioners and researchers should focus on improving support for schools to implement evidence-based programs.

## Introduction

Implementing school-based obesity prevention programs is a key public health strategy for countering the childhood obesity epidemic (1,2). In addition to establishing policies that include updated nutritional requirements for the National School Lunch Program (3), intervention programs have been developed to promote healthy eating and physical activity and prevent obesity. A recent systematic review of school-based obesity prevention interventions identified 115 programs, finding moderately strong evidence of their effectiveness (1).

As the American Academy of Pediatrics recently highlighted, a concern with obesity prevention programs is the potential for triggering unhealthy weight-control behaviors or exacerbating weight stigma (4). Adolescents with obesity are more vulnerable than those without obesity to have disordered weight-control behaviors (5–7), particularly if they are subjected to weight-related bullying (8,9). Although programs that focus on healthful eating, physical activity, and screen time habits for all students can simultaneously reduce the prevalence of obesity and disordered weight-control behaviors (10,11), programs that emphasize weight loss could be harmful (12–14).

It is unclear how broadly evidence-based programs have been disseminated and whether in the absence of effective dissemination schools instead use ineffective or even harmful programs. The Diffusion of Innovations Model (15), which suggests that exposure to knowledge about new ideas and persuasion about their adoptability are critical to adopting new programs, offers insights into the potential lag between the publication of obesity prevention research and broad adoption. Exploring processes involved in decision making about adopting evidence-based interventions can increase understanding of how to broaden their dissemination (16). We conducted a mixed-methods pilot study to evaluate how frequently schools use evidence-based obesity prevention programs; we also aimed to assess the frequency of schools that implement programs that may unintentionally exacerbate weight stigma, and we explored school administrators’ perceptions of key facilitators and barriers to adopting wellness programs. We hypothesized that evidence-based obesity prevention programs are rarely used.

## Methods

### Study design and sample

This cross-sectional pilot study was conducted from February to June 2016. To reach the target population of US public school principals, we downloaded a list of all public kindergarten through twelfth-grade schools in the United States from the National Center for Education Statistics (17) and randomly sampled from the list with the goal of recruiting 200 schools (on the basis of study resources) during the 2015–2016 school year. Principals were invited to participate and offered an incentive of entering a raffle for a $50 gift card with a one in 20 chance of winning. Another school administrator (eg, vice principal) could complete the survey if a principal felt the colleague was more knowledgeable. Of 2,387 invited principals, 247 agreed to participate or had a colleague participate (10.3% response rate). Compared with nonrespondents, respondents were from schools with significantly higher percentages of white students and significantly lower percentages of Hispanic and Asian students. Participants completed an online survey focusing on school policies related to wellness; personal information about the respondents (beyond job title) was not collected. Data were collected and managed by using REDCap electronic data capture tools hosted at the Harvard T.H. Chan School of Public Health (18). Study procedures were approved by the institutional review board at the Harvard T.H. Chan School of Public Health Office of Human Research Administration.

### Measure development

We developed a brief survey (Appendix) to gather data on school principals’ perspectives on school-based wellness and obesity prevention programs. Several closed-ended items elicited information about study participants’ priority health concerns at their schools and whether schools engaged in a broad range of wellness activities, such as having wellness policies or curricular and promotional programs. Among schools with wellness programs, the survey included questions on who was involved in choosing or developing the programs and what programs the schools used. To assess first whether the school used any predeveloped program to promote healthy nutrition, physical activity and/or screen time behaviors (rather than developing a program ad hoc), respondents were asked whether a predeveloped program was used and were supplied with a checklist of existing school wellness intervention programs from which to choose (Box) (19). From the checklist and the write-in options, the researchers then coded whether the chosen program had been evaluated in the research literature and had demonstrated evidence for effectiveness on the basis of comprehensive literature searches. Among schools that reported developing their programs onsite, we asked respondents to indicate whether the programs included a range of activities (eg, gardening, nutrition education); to assess whether the school used programs that could exacerbate weight stigma and disordered weight-control behaviors, respondents were asked whether the school used weight loss or calorie counting competitions for either students or staff or body mass index reporting for individual students (4,13). Items with Likert scale responses assessed study participants’ views on whether weight-based bullying or teasing was occurring in their schools, as well as their perception of the program’s effectiveness and impact on academic achievement, school climate, and bullying or teasing. Study participants also were asked to respond to 2 open-ended questions on their perceptions of facilitators and barriers to successfully implementing wellness programs and to provide any general comments. The survey was drafted and piloted with several school administrators and teachers, edited, and finalized.

**Box. List of Predeveloped Obesity Prevention Programs Provided to Participants**

- Brain Breaks
- bSAFE-bFIT
- Coordinated Approach to Children’s Health (CATCH)^a^
- COPE
- FOOD PLAY
- Health Ahead/Heart Smart
- Just for Kids!
- KaBoom
- KID-FIT
- New Moves^a^
- Planet Health^a^
- President’s Challenge Physical Activity and Fitness
- Project Fit America
- SPARK^a^
- Start For Life
- TAKE10^a^
- Other (write in name)

**Participants additionally wrote in:**

- Alliance for a Healthier Generation
- Eat Smart/Move More
- Build Our Kids Success (BOKS)
- Counselor-sponsored small groups
- FitnessGram
- Go Noodle
- Nutrition Network
- Safe Routes to School^a^

^a^ Programs categorized as having peer-reviewed evidence in support of the program’s efficacy.

### Statistical and qualitative analysis

We estimated the frequency of responses to each survey item for the entire sample. To explore potential disparities in access to wellness programs, we constructed 2 multivariate logistic regression models that tested whether an association existed between schools’ percentage of students receiving free or reduced-price lunch, percentage of white students, grade level, or Census region (Northeast, South, Midwest, West) and the the likelihood of 1) having any wellness programs, and 2) having an evidence-based intervention program. Analyses were conducted using Stata, Release 13 (StataCorp LP).

To analyze themes in the text written by study participants for the open-ended survey questions about facilitators or barriers for program adoption, the researchers used an immersion/crystallization approach (20). This approach to qualitative data analysis involves researchers immersing themselves in textual data (ie, reading the text repeatedly) until themes “crystallize.” At this point, researchers meet to discuss the patterns and themes that have emerged across the text. After the initial immersion process, 2 members of the study team (S.W. and E.L.K.) met to discuss broad topic categories found across the texts that could be used to organize text and allow for more detailed analysis and created a codebook for coding the textual data on the basis of these categories. Both researchers then independently coded the text submitted by each study participant by hand, finding a 98% agreement in coding. After the text data was coded and organized, these 2 investigators reviewed the text again, hand-sorted by the broad code categories, to identify important themes that emerged within and across the categories. The investigators reviewed the text until reaching consensus on a final group of key themes. Themes were analyzed first for the overall sample and then stratified based on whether or not schools 1) had an evidence-based program and 2) used a potentially stigmatizing program, to identify possible patterns in barriers related to program adoption status.

## Results

The 247 survey respondents represented public school administrators from 48 states; approximately one-sixth of respondents were from the Northeast census region (n = 41, 16.6%), a similar proportion were from the West (n = 39, 15.8%), one-third were from the South (n = 80, 32.4%), and more than one-third were from the Midwest (n = 87, 35.2%) (Table 1). Most respondents were school principals (n = 225, 91.1%); a few identified as assistant principals, nurses, or other school administrators. Forty-five percent of schools served student populations where more than 50% of students received free or reduced-price lunches.

**Table 1:** Characteristics of Schools in Survey of Public School Administrators (N = 247) on Obesity Prevention Programs, United States, 2016

Characteristic	Value^a^
**Region**	
Northeast	41 (16.6)
South	80 (32.4)
Midwest	87 (35.2)
West (includes Alaska and Hawaii)	39 (15.8)
**Respondent role**	
School principal	225 (91.1)
Assistant principal	5 (2.0)
Other, nurse, other administrator	17 (6.9)
**Percentage of students receiving free or reduced-price lunch**	
0–25	52 (21.3)
25–50	82 (33.6)
50–75	71 (29.1)
75–100	39 (16.0)
**Race/ethnicity of student body, mean % (SD)**	
Non-Hispanic white	59.5 (32.3)
Non-Hispanic black	13.8 (23.7)
Hispanic	18.8 (24.9)
Asian	2.7 (4.4)
Native Hawaiian/Pacific Islander	0.4 (2.9)
Native American/American Indian	1.8 (6.0)
Multiracial or other	3.0 (3.9)
**Estimated prevalence of obesity at school (reported by survey respondent)**	
0–25	175 (70.9)
25–50	68 (27.5)
50–75	4 (1.6)
75–100	0

Abbreviation: SD, standard deviation.

a Values expressed as no. (%) unless otherwise indicated.

A minority of survey respondents ranked obesity, nutrition, or physical activity as among their top 3 health concerns for their school populations (Table 2). Eighty-five participants (34.4%) ranked physical inactivity as one of their top 3 student health concerns, 65 (26.3%) listed nutrition, and 42 (17.0%) listed overweight and obesity. In contrast, emotional and mental health was ranked as a top concern by 81.4% of respondents, and relational/social skills were ranked by 67.2% respondents. One-fifth of participants (n = 53, 21.5%) reported that weight-related teasing and bullying was a problem.

**Table 2:** School Administrators’ Implementation of Obesity Prevention Programs and Perceptions of Impact, United States, 2016

Characteristic	No. (%) of Participants Reporting (N = 247)	% of Participants With Obesity Prevention Activities in Place (n = 117)
**Obesity ranked as one of top 3 health concerns at school**	42 (17.0)	—
**Nutrition ranked as one of top 3 health concerns at school**	65 (26.3)	—
**Physical inactivity ranked as one of top 3 health concerns at school**	85 (34.4)	—
**Weight-related teasing and/or bullying is “somewhat of a problem” or a “significant problem”**	53 (21.5)	—
**School offers wellness or obesity prevention activities**	117 (47.4)	—
For all grades	88 (35.6)	—
For some grades only	29 (11.7)	—
**School uses any predeveloped wellness or obesity prevention intervention program**	17 (6.9)	14.5
**School uses an evidence-based obesity prevention program**	7 (2.8)	6.0
**School developed its own obesity prevention program(s) onsite**	70 (28.3)	59.8
**Focus of onsite program(s)**		
Physical education	66 (26.7)	94.3
Nutrition education and promotion	53 (21.5)	75.7
Physical activity outside of physical education	47 (19.0)	67.1
Nutrition standards for school meals	46 (18.6)	65.7
Weight loss competition — staff	28 (11.3)	40.0
BMI tracking — individual	18 (7.3)	25.7
Standards for competitive foods	18 (7.3)	25.7
School garden	16 (6.5)	22.9
Program monitoring, evaluation, and reporting	11 (4.5)	15.7
Collaboration with students and families	9 (3.6)	12.9
BMI tracking — overall school reporting	6 (2.4)	8.6
Calorie counting competition	5 (2.0)	7.1
Other: after school sports and fitness	1 (0.4)	1.4
Other: health class required	1 (0.4)	1.4
Weight loss competition — students	0	0
**Perceived overall impact of obesity prevention program^a^**		
Very or generally successful	47 ( — )	41.6
Marginally successful	42 ( — )	37.2
Neither successful nor unsuccessful	17 ( — )	15.0
Unsuccessful	7 ( — )	6.2

Abbreviations: BMI, body mass index.

a Of the 113 participants with obesity prevention activities in place and who answered question.

Fewer than half of respondents (n = 117, 47.4%) indicated that their schools offered a school wellness or obesity prevention activity of any kind (including broad policies such as competitive foods standards and more structured programs) (Table 2). Although several respondents indicated that their schools had worked to improve the food environment through standards for school meals (n = 46, 18.6%) and competitive foods (n = 18, 7.3%), and many schools reported using physical education (n = 66, 26.7%) or nutrition education of some kind (n = 53, 21.5%), only 17 respondents (6.9%) reported using a predeveloped program focused on nutrition or physical activity, and only 7 (2.8%) reported using a program with research-based evidence for effectiveness; programs used included the Coordinated Approach to Child Health (CATCH) (21), Sports, Play, and Active Recreation for Kids (SPARK) (22), TAKE10 (23), and Safe Routes to School (24) programs. School programs that focused on student or staff weight, rather than on healthy eating and physical activity behaviors, were much more frequently used. Twenty-eight (11.3%) schools reported using weight loss competitions for staff, 5 (2.0%) schools reported using calorie counting competitions, and 18 (7.3%) schools reported using individual body mass index tracking.

Among the 117 schools that reported implementing any type of wellness and obesity prevention activity, survey respondents, using a checklist of potential partners in decision making, ranked physical education teachers (n = 77, 65.8%) most frequently as people involved in the program development or selection process. Participants whose schools offered obesity prevention activities indicated that recommendations from a district administrator (n = 32, 27.4%), the fact that other schools in the district were using the same program (n = 28, 23.9%), and recommendation from a teacher at their own school (n = 28, 23.9%) were common factors in programming decisions. A handful of participants at schools with obesity prevention activities reported that their school programming decisions were influenced by reviews of educational (n = 12, 10.3%) or scientific (n = 6, 5.1%) literature. More than half of administrators at schools with wellness activities (n = 64, 54.7%) perceived a positive impact of their activities on overall student health, and nearly two-thirds (n = 72, 61.5%) perceived a positive impact on student physical health. School administrators generally perceived that their wellness programs did not increase eating disorders (n = 97, 95.1%). Thirty-eight (32.5%) participants thought that their programming led to a decrease in unhealthy weight management by students.

Results from the logistic regression models testing whether the school’s region or sociodemographic makeup predicted likelihood of having 1) any wellness program or 2) an evidence-based intervention suggested that none of these variables (school’s percentage of students receiving free or reduced-price lunch, percentage of white students, grade level, and Census region (Northeast, South, Midwest, West) were significantly associated with either outcome.

Most survey respondents (n = 232, 93.9%) offered commentary in response to open-ended questions about barriers and supports to implementing obesity prevention or nutrition and physical activity programs. Across schools, respondents frequently mentioned financial resources, time and curricular priorities, and staffing and training as key factors in determining uptake of a wellness program; some noted that financial resources and training opportunities were instrumental in facilitating the program, and others reported that a lack of these resources prohibited program adoption. One administrator noted, “Being grant-funded [by the state], our available resources are extremely limited. Once classrooms are staffed and materials for teaching purchased for core subject areas, very little is left,” echoing the comments of many administrators whose schools were lacking funding, staffing, and time to focus on wellness and obesity prevention. Others described district-level coordination and collaboration, and state and federal standards and requirements, as key structural facilitators and barriers to the success of their wellness programming

Stratifying the qualitative data by whether the school used any kind of predeveloped program, we found that respondents from schools with such programs were more likely to raise the themes of having an important program champion, substantial buy-in for the program within the school community, or both.

## Discussion

In this study of a national random sample of US public primary and secondary schools, we found that, despite the substantial amount of scientific research devoted to developing and testing school-based obesity prevention and nutrition or physical activity promotion programs (1), very few schools — less than 3% — reported implementing such programs, and less than half of schools reported opting to implement any kind of nutrition, physical activity, or obesity prevention strategy at all. Among schools that are trying to implement obesity prevention or school wellness programs, most appear to be developing programs on their own. This finding is concerning, both because school staff should not be obligated to devote scarce time and resources to developing programs when programs already exist and because well-intentioned school staff may be instituting programs that are ineffective and even potentially harmful.

In our sample of US kindergarten through twelfth-grade public schools, programs emphasizing weight loss were more commonly used than evidence-based, effective programs for promoting healthy eating and physical activity. Although programs that focus on improved nutrition, physical activity, and screen time behaviors generally prevent obesity and improve health, programs that focus instead on weight status and weight loss can exacerbate both weight stigma and unhealthy weight-control behaviors (4). Particularly concerning are staff weight-loss competitions in the style of the television show “The Biggest Loser.” The show has been associated with increasing individuals’ negatively biased attitudes toward people with obesity (25).

The themes that emerged from survey respondents’ comments about school wellness programs also suggest that schools need more support to adopt effective programs. Schools that had adopted any program, whether evidence-based or not, were more likely to describe as key facilitators having a program champion and adequate buy-in from stakeholders; a prior study of the postresearch sustainability of one evidence-based program, “New Moves,” also found buy-in, funding, and time to be critical implementation factors (26). These findings were consistent with a Diffusion of Innovations framework, suggesting that opinion leadership and change readiness based on perceived advantages and compatibility with existing systems are essential for uptake of new programs (15). However, most schools clearly are not receiving adequate information about effective programs, despite having other critical elements for program adoption in place, and thus appear to be using ineffective programs instead. In addition to improving schools’ access to communication channels about effective programs, several other adoption factors were raised by respondents. Across schools, regardless of whether they had implemented a program, respondents cited funding as a key factor; they also cited access to adequate training opportunities for staff and having structured support for programming, both within schools and at higher levels of school administration (eg, the school district, the state education agency). Taken together, these findings suggest that schools need support from public health professionals in learning about and choosing effective programs and accessing support for training and technical assistance. Public health agencies could consider partnering directly with education agencies to provide the support and expertise necessary to help schools implement effective, evidence-based programs and potentially to support “de-implementation” of existing ineffective or stigmatizing programs (27). De-implementation may be a critical effort, given that most respondents reported that their programs were effective, even in the absence of evidence.

Strengths of this study are its random sampling approach, which reduces sampling bias, and its use of mixed methods to gather a nuanced picture of school-based wellness programming. The study also has several limitations. Although a random sample allowed for a broad cross-section of US schools, we found that participating schools had slightly higher percentages of white students than did nonparticipating schools, suggesting that the results may not be generalizable to all US public schools. Schools that responded to our survey may have also differed from nonresponding schools in other ways (such as having more resources), raising the risk of response bias. Given study resource constraints, our sample was also small, raising the risk of error in our estimates. However, the margin of error for our findings on the percentage of schools using evidence-based programs was 3.2%, indicating that the study’s overall conclusions about low levels of dissemination of these programs are likely generalizable. Our small sample size may have precluded us from detecting regional or sociodemographic differences; future research, using larger samples, should further evaluate possible predictors of dissemination. The sample also did not include private or parochial schools. Additionally, the study relied on school administrators’ reports of school wellness programming, the validity of which are unknown; it is possible that respondents may have either overestimated the efficacy of wellness programs at their schools or overlooked them entirely or that school principals may have been unaware of some programs implemented in their school. Future research could compare school policies and curricular materials with school administrator reports to ascertain their accuracy.

Few schools in this study implemented nutrition, physical activity, or obesity prevention programs that known to be effective. In the absence of evidence, many schools appear to be trying to address obesity prevention and wellness on their own, unintentionally implementing potentially ineffective or harmful programs. Public health agencies and funding bodies should focus on supporting schools in the dissemination and adoption of safe and effective programs for promoting healthy eating and physical activity and preventing obesity.

## Appendix Survey Items, Survey of Public School Administrators on Obesity Prevention Programs, United States, 2016

Q1. What is your job title at the school where you work? In which role do you spend more time?

- Principal
- Assistant Principal
- Other school administrator or teacher (write in)

Q2. What do you think are the top three health concerns for your student population? Choose from list below.

- Alcohol and/or drug use
- Cognitive development
- Chronic and communicable diseases
- Emotional and mental health
- Physical fitness/physical inactivity
- Nutrition
- Overweight/obesity
- Relational and social skills
- Safety (ie, injuries)
- Sexual health
- Tobacco use
- Violence

Q3. What is the approximate proportion of students in your school who are overweight or obese? It’s OK to estimate if you are unsure of exact numbers.

- 0%–25%
- 25%–50%
- 50%–75%
- >75%

Q4. What is the approximate proportion of students in your school who you think might have eating disorders? It’s OK to estimate if you are unsure of exact numbers.

- 0%–25%
- 25%–50%
- 50%–75%
- >75%

Q5-6. How much of a problem are the following issues at your school?

- Teasing and bullying, in general:
  Not sure
  Not at all
  Somewhat of a problem
  Significant problem
- Weight-related teasing and bullying:
  Not sure
  Not at all
  Somewhat of a problem
  Significant problem
- School-Based Wellness and Obesity Prevention Programming

Q7. Does your school offer any school-based wellness or obesity prevention programs?

- No
- Yes, for all grades
- Yes, for some grades (continue to next question)

Which grades at your school have school-based wellness or obesity prevention programs? (please check all that apply)

- JK/Pre-K
- K
- 1
- 2
- 3
- 4
- 5
- 6
- 7
- 8
- 9
- 10
- 11
- 12

Q8. (If yes to Q7) Does your school have a packaged or predeveloped school-based wellness or obesity prevention program(s)?

- No
- Yes

(If yes to Q8) Name of program:

- Brain Breaks
- bSAFE-bFIT
- Coordinated Approach to Children’s Health (CATCH)
- COPE
- FOOD PLAY
- Health Ahead/Heart Smart
- Just for Kids!
- KaBoom
- KID-FIT
- New Moves
- Planet Health
- President’s Challenge Physical Activity and Fitness
- Project Fit America
- SPARK
- Start For Life
- TAKE 10!
- Other (write in name)

Q9. (If yes to Q7) Does your school offer wellness or obesity prevention programs developed by you or others at the school?

- No
- Yes

(If yes to Q9) Program(s) addressing (check all that apply):

- BMI tracking and reporting for individual students (“BMI report cards”)
- BMI tracking and reporting for the school overall (eg, a school average)
- Calorie counting competition
- Collaboration with students and families
- Nutrition education and promotion
- Nutrition standards for school meals
- Physical activity outside of physical education
- Physical education
- Program monitoring, evaluation, and reporting
- School garden
- Standards for foods offered outside of school meals (“competitive foods”)
- Weight loss competition among staff
- Weight loss competition among students
- Other (please write in)

Q10. (If yes to Q7) Who was involved in the process of selecting or developing wellness or obesity prevention programs currently offered at your school?

- Principal
- Assistant Principal
- School Nurse
- School Guidance Counselor
- Other Administrators
- Health Teachers
- Physical Education Teachers
- Other Teachers
- School Staff
- Students
- Parents
- School District Administrators
- District School Committee
- Other (please write in)

Q11. (If yes to Q7) How did you or others select the school-based wellness or obesity prevention program(s) currently offered at your school? Please check all that apply.

- Other schools in the district use the same program(s)
- District administrator recommended the program
- School administrator recommended the program
- Teacher at this school recommended the program
- Member of the school community other than a teacher or administrator recommended the program
- Program was identified from a review of scientific literature
- Program was identified from a review of education literature
- Grant-funding specified adoption of the program
- Other (please write in)

Q12. (If yes to Q7) How successful do you feel your school-based wellness or obesity prevention program(s) has been?

- Very successful
- Generally successful
- Marginally successful
- Neither successful nor unsuccessful
- Marginally unsuccessful
- Generally unsuccessful
- Very unsuccessful

Q13. (If yes to Q7) On average, how do you think the wellness or obesity prevention programs currently offered by your school are impacting students in the following areas (response options: none, neutral, positive):

- Academic achievement
- Overall health
- Physical health
- Mental health

Q14. (If yes to Q7) Do you think the school-based wellness or obesity prevention programs currently offered by your school have increased, decreased, or had no impact on the following (response options: increased, no impact, decreased):

- Bullying
- Eating disorders
- Parent engagement
- Student engagement
- Staff stress levels
- Student stress levels
- Student–teacher communication
- Teacher–parent communication
- Teacher satisfaction
- Teasing based on appearance
- Teasing based on weight
- Teasing based on other factors
- Unhealthy weight management behaviors

Q15. (If yes to Q7) How have you evaluated the success of your school-based wellness or obesity prevention program(s)?

- Data collected from students
- Data collected from teachers
- Observation of students
- Discussion with students
- Discussion with faculty and staff
- Discussion with parents and/or community stakeholders
- Not currently measuring program impacts

Q16. What factors have impacted the success or failure of your school’s wellness or obesity prevention program(s)? If you do not currently have any school-based wellness or obesity prevention programs, then what factors have impacted the decision not to have these types of programs? (Open-ended)

Q17. Your comments on school-based wellness and obesity prevention programming: (Open-ended)
